# Crystal structure and Hirshfeld surface analysis of 2,2,2-tri­fluoro-1-(7-methyl­imidazo[1,2-*a*]pyridin-3-yl)ethan-1-one

**DOI:** 10.1107/S2056989021012676

**Published:** 2022-01-01

**Authors:** Firudin I. Guseinov, Konstantin I. Kobrakov, Bogdan I. Ugrak, Zeliha Atioğlu, Mehmet Akkurt, Ajaya Bhattarai

**Affiliations:** a Kosygin State University of Russia, 117997 Moscow, Russian Federation; b N. D. Zelinsky Institute of Organic Chemistry, Russian Academy of Sciences, 119991 Moscow, Russian Federation; c Department of Aircraft Electrics and Electronics, School of Applied Sciences, Cappadocia University, Mustafapaşa, 50420 Ürgüp, Nevşehir, Turkey; d Department of Physics, Faculty of Sciences, Erciyes University, 38039 Kayseri, Turkey; e Department of Chemistry, M.M.A.M.C (Tribhuvan University) Biratnagar, Nepal

**Keywords:** crystal structure, imidazo[1,2-*a*]pyridine, hydrogen bonds, F⋯F contacts, Hirshfeld surface analysis

## Abstract

The bicyclic imidazo[1,2-*a*]pyridine core in the mol­ecule of the title compound, C_10_H_7_F_3_N_2_O, is planar within 0.004 (1) Å. In the crystal, the mol­ecules are linked by pairs of C—H⋯N and C—H⋯O hydrogen bonds, forming strips. These strips are connected by the F⋯F contacts into layers, which are further joined by π–π stacking inter­actions. The Hirshfeld surface analysis and fingerprint plots reveal that mol­ecular packing is governed by F⋯H/H⋯F (31.6%), H⋯H (16.8%), C⋯H/H⋯C (13.8%) and O⋯H/H⋯O (8.5%) contacts.

## Abstract 2

In the crystal, the mol­ecules are linked by C—H⋯N and C—H⋯O hydrogen bonds into strips, which are connected by F⋯F contacts into layers.

## Chemical context

The imidazo[1,2-*a*]pyridine synthon is one of the important fused bicyclic 5–6 heterocycles and it is recognized as a ‘drug prejudice’ scaffold because of its wide range of applications in medicinal chemistry (Bagdi *et al.*, 2015 ▸). This synthon is also useful in coordination chemistry and catalysis because of its coordination ability and non-covalent donor–acceptor bonding (Guseinov *et al.*, 2022 ▸; Ma *et al.*, 2020 ▸, 2021 ▸; Mahmudov *et al.*, 2020 ▸, 2021 ▸). Synthesis of this synthon from easily available chemicals is desirable due to its importance in the various branches of chemistry (Bagdi *et al.*, 2015 ▸). Along with this, inter­molecular inter­actions organize mol­ecular architectures, which play a crucial role in synthesis, catalysis, micellization, *etc.* (Gurbanov *et al.*, 2020*a* ▸,*b* ▸; Kopylovich *et al.*, 2011 ▸; Ma *et al.*, 2017*a* ▸,*b* ▸). The non-covalent bond–acceptor ability of both nitro­gen atoms in the imidazo[1,2-*a*]pyridine synthon can be used in crystal engineering and in the design of dyes and other materials (Maharramov *et al.*, 2018 ▸; Mizar *et al.*, 2012 ▸; Shixaliyev *et al.*, 2014 ▸; Shikhaliyev *et al.*, 2018 ▸, 2019 ▸). Herein, we report a one-pot synthesis of 2,2,2-tri­fluoro-1-(7-methyl­imidazo[1,2-*a*]pyridin-3-yl)ethan-1-one (I) from (*E*/*Z*)-3-bromo-1,1,1-tri­fluoro-4-isopropoxybut-3-en-2-one and 4-methyl­pyridin-2-amine, which provides multiple inter­molecular non-covalent inter­actions. 
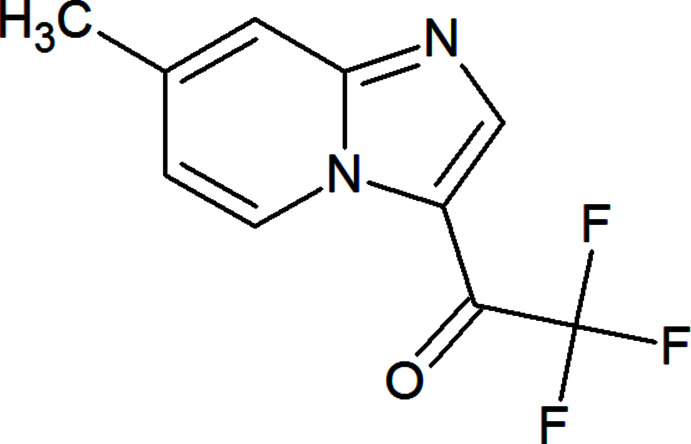


## Structural commentary

**Figure 1:**
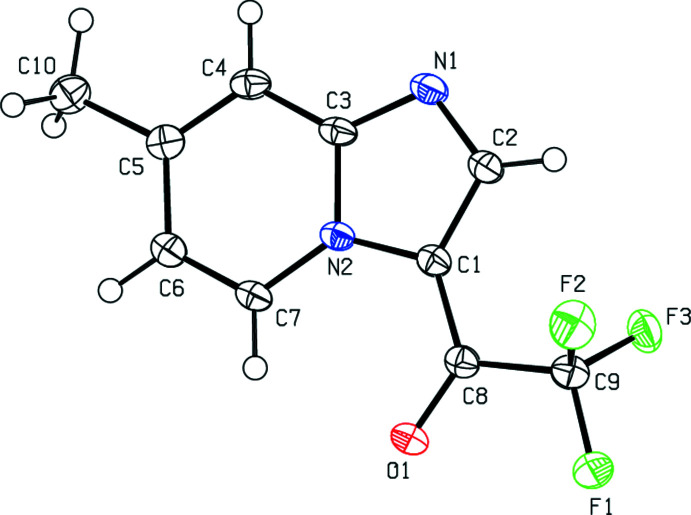
Mol­ecular structure of the title compound showing the atom labelling and displacement ellipsoids drawn at the 50% probability level.

In the mol­ecule of the title compound (Fig. 1 ▸), the fused bicyclic imidazo[1,2-*a*]pyridine core is planar within 0.004 (1) Å, with a dihedral angle of 0.34 (6)° between the mean planes of the five- and six-membered rings. The C2—C1—C8—C9 and N2—C1—C8—O1 torsion angles of 1.04 (18) and 1.14 (19)°, respectively, show that the ethanone group lies near the plane of the bicycle. The bond lengths N1—C2, C2—C1 and C1—C8 of 1.3367 (16), 1.3987 (16) and 1.4247 (16) Å, respectively, indicate strong π-conjugation in the N1–O1 chain.

## Supra­molecular features and Hirshfeld surface analysis

**Figure 2:**
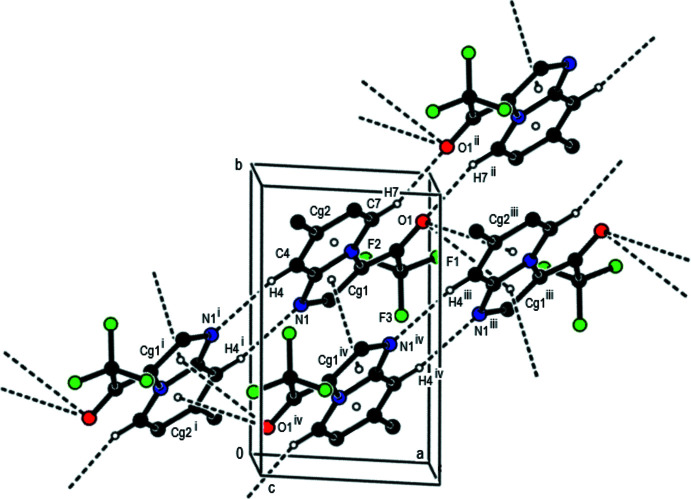
A general view of the C—H⋯O and C—H⋯N hydrogen bonds and π–π stacking inter­actions in the title compound, depicted by dashed lines. Hydrogen atoms not involved in hydrogen bonding are omitted. [Symmetry codes: (i) −*x*, −*y* + 1, −*z* + 1; (ii) −*x* + 2, −*y* + 2, −*z* + 1; (iii) *x* + 1, *y*, *z*; (iv) −1 + *x*, *y*, *z*.]

**Figure 3:**
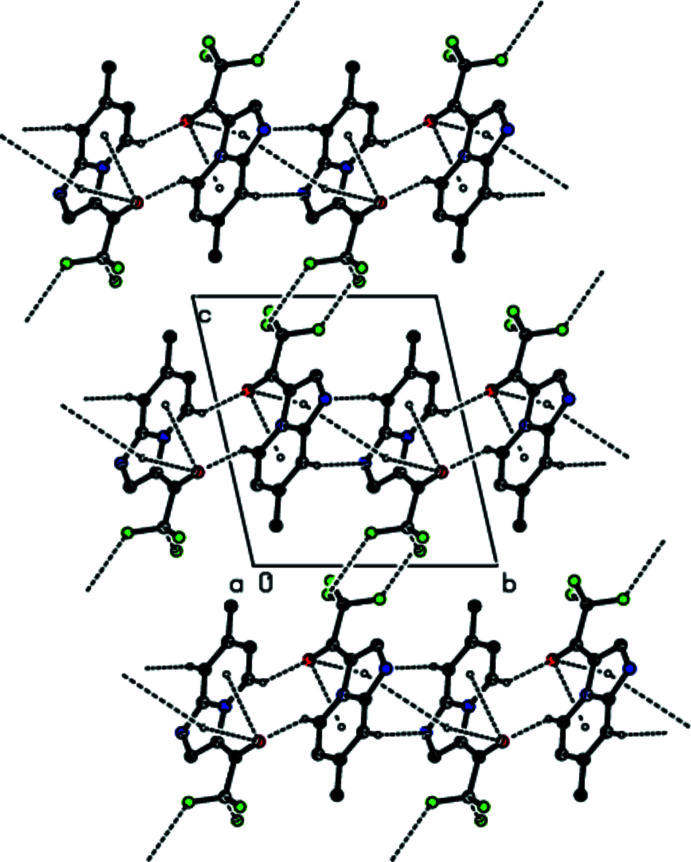
Packing diagram of the title compound, viewed down the *a* axis showing the C—H⋯O and C—H⋯N hydrogen bonds and the F⋯F and π–π stacking inter­actions. Hydrogen atoms not involved in hydrogen bonding are omitted.

**Figure 5:**
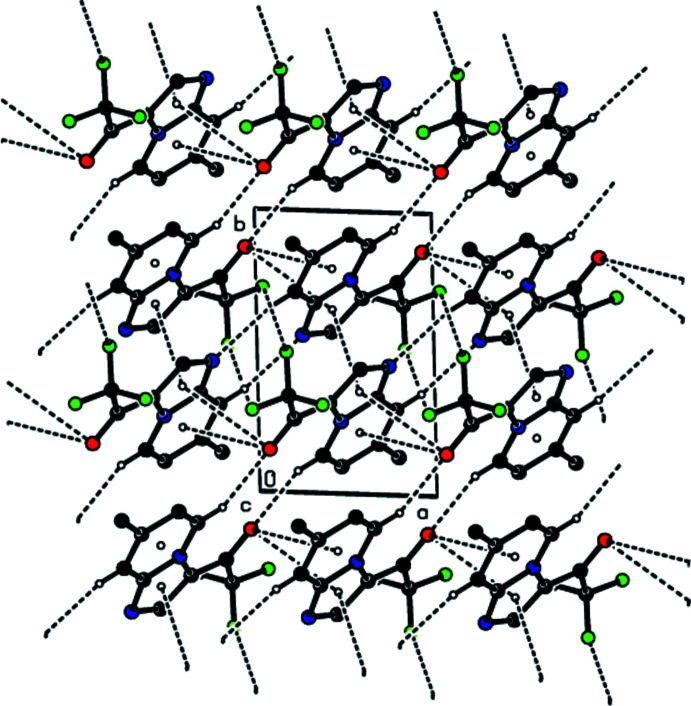
Packing diagram of the title compound, viewed down the *c* axis showing the C—H⋯O and C—H⋯N hydrogen bonds and the F⋯F and π–π stacking inter­actions. Hydrogen atoms not involved in hydrogen bonding are omitted.

In the crystal, the mol­ecules are linked by pairs of C—H⋯N and C—H⋯O hydrogen bonds into strips elongated along the [210] direction (Figs. 2 ▸ and 3 ▸, Table 1 ▸). These strips are joined into layers parallel to (1 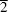 2) by F⋯F contacts (Figs. 3 ▸–5 ▸ ▸, Table 2 ▸). The layers are connected by F⋯H contacts (Fig. 5 ▸, Table 2 ▸) and π–π inter­actions with a shortest inter­centroid separation of 3.6395 (7) Å [*C*g1⋯*Cg*1(1 − *x*, 1 − *y*, 1 − *z*); *Cg*1 is the centroid of the imidazole ring].

**Figure 6:**
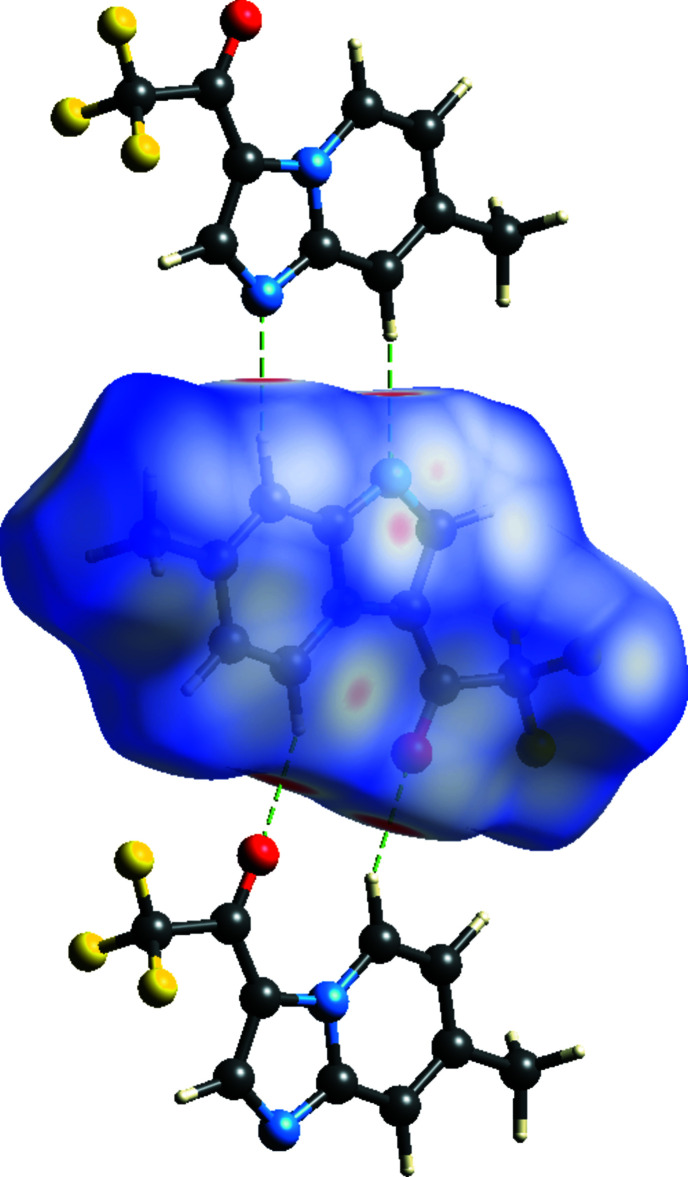
Hirshfeld surface of the title mol­ecule mapped over *d*_norm_.

**Figure 7:**
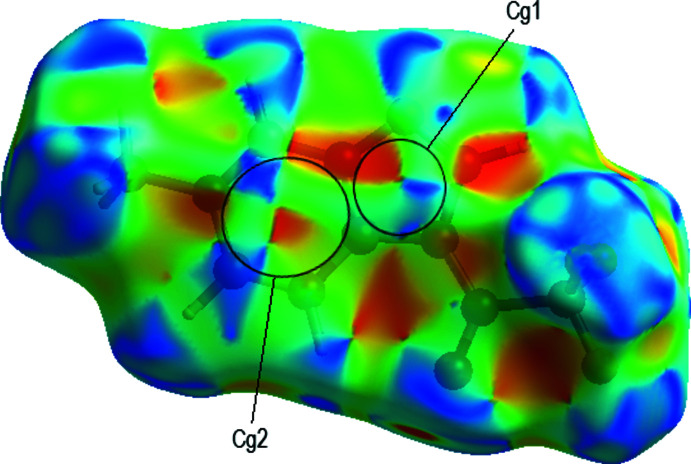
Hirshfeld surface of the title compound plotted over shape-index.

**Figure 8:**
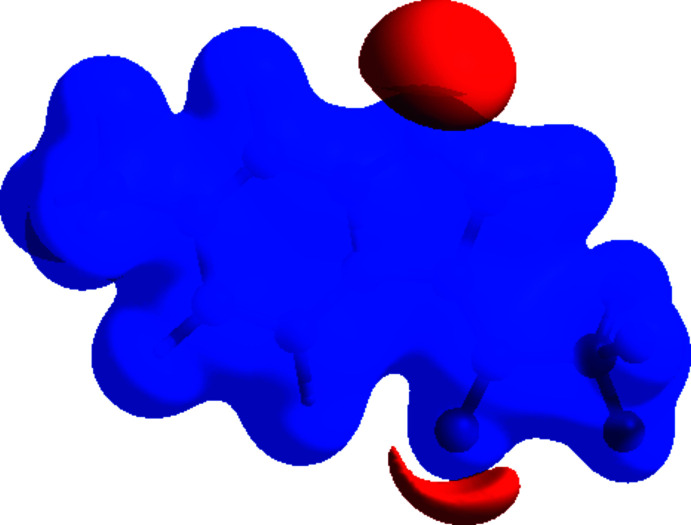
View of the three-dimensional Hirshfeld surface of the title mol­ecule plotted over electrostatic potential energy in the range −0.0500 to 0.0500 a.u. calculated at the Hartree–Fock level of theory using the *STO-3 G* basis set. Hydrogen-bond donors and acceptors are shown as blue and red regions around the atoms, corresponding to positive and negative potentials, respectively.

**Figure 9:**
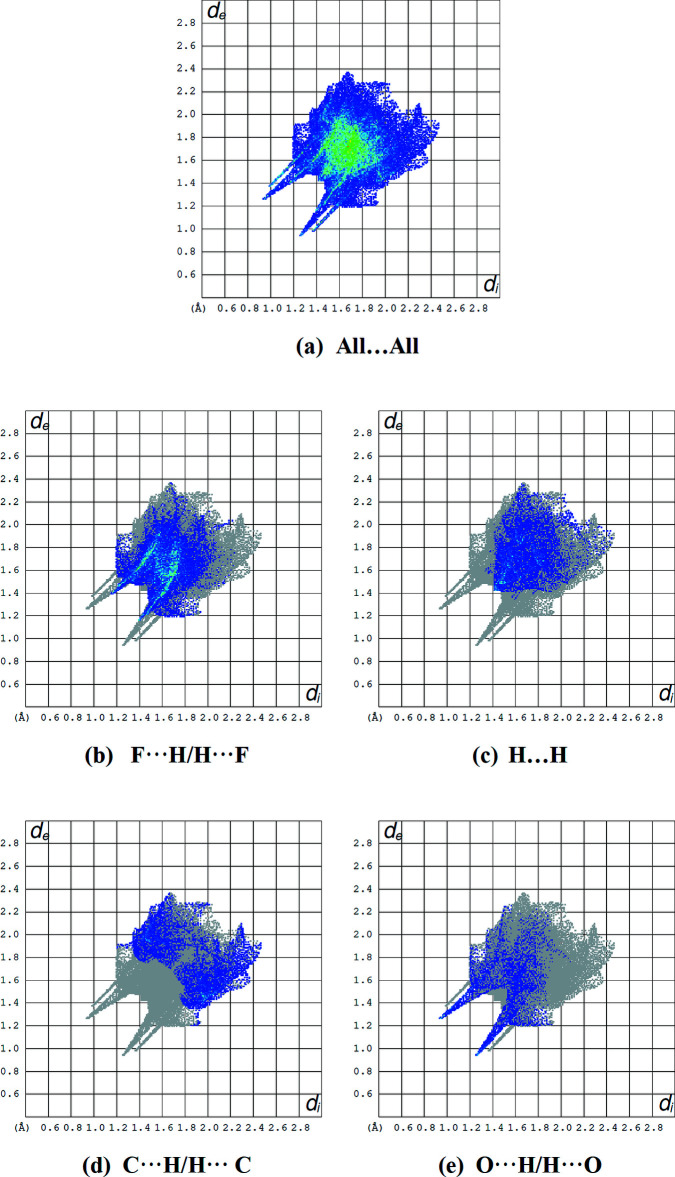
(*a*) The overall two-dimensional fingerprint plot and those delineated into (*b*) F⋯H/H⋯F, (*c*) H⋯H, (*d*) C⋯H/H⋯C and (*e*) O⋯H/H⋯O inter­actions.

To visualize the inter­molecular inter­actions in the title compound, the 3D Hirshfeld surfaces and two-dimensional fingerprint plots were computed using *Crystal Explorer 17* (Turner *et al.*, 2017 ▸). The Hirshfeld surface plotted over *d*_norm_ in the range −0.3137 to 1.1314 a.u. is shown in Fig. 6 ▸. The intense red spots with negative *d*_norm_ values represent C—H⋯O and C—H⋯N hydrogen bonds. Pale red spots correspond to π–π inter­actions, which are also seen in the shape-index surface (Fig. 7 ▸) generated in the range −1 to 1 Å, where they are indicated by adjacent red and blue triangles. The Hirshfeld surface mapped over the electrostatic potential is shown in Fig. 8 ▸, where the hydrogen-bond acceptors are represented as red regions. The overall two-dimensional fingerprint plot, and those delineated into F⋯H/H⋯F (31.6%), H⋯H (16.8%), C⋯H/H⋯C (13.8%) and O⋯H/H⋯O (8.5%) contacts are illustrated in Fig. 9 ▸. Other minor contributions to the Hirshfeld surface are from N⋯H/H⋯N (7.7%), F⋯F (6.1%), O⋯C/C⋯O (4.2%), N⋯C/C⋯N (3.8%), C⋯C (2.4%), F⋯C/C⋯F (1.7%), F⋯N/N⋯F (1.4%), N⋯N (1.1%) and O⋯N/N⋯O (0.9%) contacts.

## Database survey

The most closely related compounds containing a similar imidazo[1,2-*a*]pyridine skeleton, but with different substituents on the amide N atom are: *N*-*t*-butyl-2-(phenyl­ethyn­yl)imidazo[1,2-*a*]pyridin-3-amine (XOWVOX; Tber *et al.*, 2019 ▸), 6-bromo-2-(4-bromo­phen­yl)imidazo[1,2-*a*]pyridine (KOXGEM; Khamees *et al.*, 2019 ▸), *N*-*t*-butyl-2-(2-nitro­phen­yl)imidazo[1,2-*a*]pyridin-3-amine (PILGAV01; Dhanalakshmi *et al.*, 2019 ▸), 2-(4-meth­oxy­phen­yl)-6-nitro­imidazo[1,2-*a*]pyridine-3-carbaldehyde (DABTEI; Koudad *et al.*, 2015 ▸), 2-(ethyl­sulfin­yl)imidazo[1,2-*a*]pyridine-3-sulfonamide (ZAP­JAD; Gong *et al.*, 2012 ▸) and 2-methyl-6-(tri­fluoro­meth­yl)imidazo[1,2-*a*]pyridine-3-carbo­nitrile (ULEGOI; Fun *et al.*, 2011 ▸). In the crystal of XOWVOX, mol­ecules are linked by N—H⋯H hydrogen bonds, forming chains along the *c*-axis direction. The chains are linked by C—H⋯π inter­actions, forming slabs parallel to the *ac* plane. In the structure of KOXGEM, an intra­molecular C—H⋯N hydrogen bond forms an *S*(5) ring motif. In the crystal, a short H⋯H contact links adjacent mol­ecules into centrosymmetric dimers. The dimers are joined by weak C—H⋯π and slipped π–π stacking inter­actions, forming layers parallel to (110), which are connected into a three-dimensional network by short Br⋯H contacts. In the crystal of PILGAV01, N—H⋯N hydrogen bonds link the mol­ecules into [010] chains. The cohesion of the crystal structure of DABTEI is ensured by C—H⋯N and C—H⋯O hydrogen bonds, forming layers parallel to the *ac* plane. In ZAPJAD, the supra­molecular structure is defined by two kinds of inter­molecular hydrogen bonds. Pairs of N—H⋯N hydrogen bonds link the mol­ecules into centrosymmetric dimers and N—H⋯O hydrogen bonds link the dimers into tubular chains running along the *a-*axis direction. In the crystal of ULEGOI, mol­ecules are linked into chains through pairs of C—H⋯N inter­actions, forming 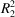 (12) and 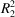 (8) hydrogen-bond ring motifs. These chains are stacked along the *a* axis.

## Synthesis and crystallization

A mixture of (*E*/*Z*)-3-bromo-1,1,1-tri­fluoro-4-isopropoxybut-3-en-2-one (0.522 mg, 2 mmol) and 4-methyl­pyridin-2-amine (0.216 mg, 2 mmol) in dry isopropyl alcohol (15 mL) was refluxed for 4 h. Then the solvent was removed on a rotary evaporator under reduced pressure. The residue was recrystallized from methanol. Crystals suitable for X-ray analysis were obtained by slow evaporation of a methanol solution. Colourless solid (yield 94%), m.p. 405–406 K. Analysis calculated for C_10_H_7_F_3_N_2_O (*M* = 228.17): C 52.64, H 3.09, N 12.28; found: C 52.55, H 3.07, N 12.19%. ^1^H NMR (300 MHz, CDCl_3_) *δ* 2.48 (3H, CH_3_), 7.32–8.60 (3H, Ar), 9.32 (1H, CH). ^13^C NMR (75 MHz, CDCl_3_) δ 174.45, 150.22, 141.32, 135.67, 131.04, 123.55, 118.66, 118.21, 116.32 and 21.56. ESI–MS: *m*/*z*: 229.18 [*M* + H]^+^.

## Refinement

Crystal data, data collection and structure refinement details are summarized in Table 3 ▸. Hydrogen atoms were positioned geometrically (C—H = 0.95–0.98 Å) and refined as riding, with *U*_iso_(H) = 1.2*U*_eq_(C) for CH hydrogen atoms and *U*_iso_(H) = 1.5*U*_eq_(C) for CH_3_ hydrogen atoms.

## Supplementary Material

Crystal structure: contains datablock(s) I. DOI: 10.1107/S2056989021012676/yk2161sup1.cif

Structure factors: contains datablock(s) I. DOI: 10.1107/S2056989021012676/yk2161Isup2.hkl

Supporting information file. DOI: 10.1107/S2056989021012676/yk2161Isup3.cml

CCDC reference: 2124974

Additional supporting information:  crystallographic information; 3D view; checkCIF report

## Figures and Tables

**Figure 4:**
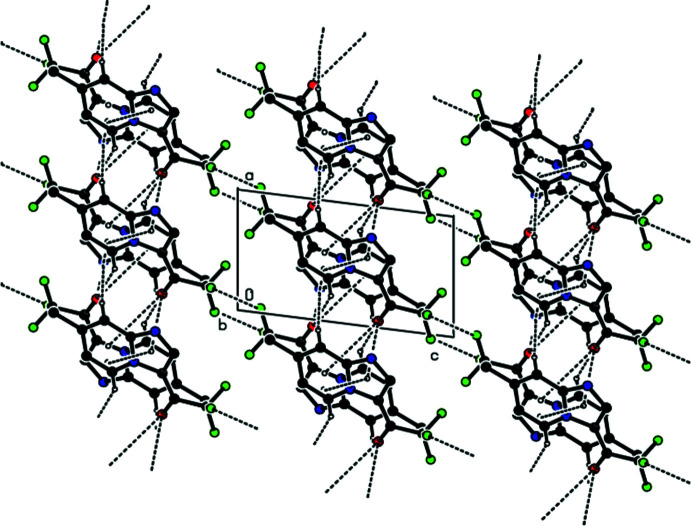
Packing diagram of the title compound, viewed down the *b* axis showing the C—H⋯O and C—H⋯N hydrogen bonds and the F⋯F and π–π stacking inter­actions. Hydrogen atoms not involved in hydrogen bonding are omitted.

**Table 1:** Hydrogen-bond geometry (Å, °)

*D*—H⋯*A*	*D*—H	H⋯*A*	*D*⋯*A*	*D*—H⋯*A*
C4—H4⋯N1^i^	0.95	2.48	3.4139 (16)	167
C7—H7⋯O1^ii^	0.95	2.30	3.1464 (14)	147

Symmetry codes: (i) -x, -y+1, -z+1; (ii) -x+2, -y+2, -z+1.

**Table 2:** Summary of short inter­atomic contacts (Å) in the title structure

Contact	Distance	Symmetry operation
O1⋯C3	3.1574 (15)	1 + *x*, *y*, *z*
F1⋯H10*B*	2.86	1 + *x*, *y*, −1 + *z*
F3⋯F1	2.9074 (11)	2 − *x*, 1 − *y*, −*z*
H10*C*⋯O1	2.83	1 − *x*, 2 − *y*, 1 − *z*
F2⋯H10*A*	2.64	*x*, *y*, −1 + *z*
H2⋯F2	2.80	1 − *x*, 1 − *y*, −*z*
C3⋯N1	3.3055 (16)	1 − *x*, 1 − *y*, 1 − *z*

**Table 3:** Experimental details

Crystal data	
Chemical formula	C_10_H_7_F_3_N_2_O
*M*_r_	228.18
Crystal system, space group	Triclinic, *P*\overline{1}
Temperature (K)	100
*a*, *b*, *c* (Å)	5.4384 (1), 8.8298 (2), 10.0744 (2)
α, β, γ (°)	102.501 (2), 96.764 (2), 91.415 (2)
*V* (Å^3^)	468.39 (2)
*Z*	2
Radiation type	Cu *K*α
μ (mm^−1^)	1.30
Crystal size (mm)	0.15 × 0.06 × 0.02

Data collection	
Diffractometer	XtaLAB Synergy, Dualflex, HyPix
Absorption correction	Multi-scan (*CrysAlis PRO*; Rigaku OD, 2021 ▸)
*T*_min_, *T*_max_	0.323, 1.000
No. of measured, independent and observed [*I* > 2σ(*I*)] reflections	14165, 2006, 1927
*R*_int_	0.050
(sin θ/λ)_max_ (Å^−1^)	0.638

Refinement	
*R*[*F* ^2^ > 2σ(*F* ^2^)], *wR*(*F* ^2^), *S*	0.037, 0.105, 1.09
No. of reflections	2006
No. of parameters	147
H-atom treatment	H-atom parameters constrained
Δρ_max_, Δρ_min_ (e Å^−3^)	0.37, −0.26

Computer programs: *CrysAlis PRO* (Rigaku OD, 2021 ▸), *SHELXT* (Sheldrick, 2015*a* ▸), *SHELXL* (Sheldrick, 2015*b* ▸), *ORTEP-3 for Windows* (Farrugia, 2012 ▸) and *PLATON* (Spek, 2020 ▸).

## supplementary crystallographic information

### Crystal data

**Table:**

C_10_H_7_F_3_N_2_O	*Z* = 2
*M**_r_* = 228.18	*F*(000) = 232
Triclinic, *P*1	*D*_x_ = 1.618 Mg m^−^^3^
*a* = 5.4384 (1) Å	Cu *K*α radiation, λ = 1.54184 Å
*b* = 8.8298 (2) Å	Cell parameters from 11472 reflections
*c* = 10.0744 (2) Å	θ = 4.5–79.1°
α = 102.501 (2)°	µ = 1.30 mm^−^^1^
β = 96.764 (2)°	*T* = 100 K
γ = 91.415 (2)°	Block, colorless
*V* = 468.39 (2) Å^3^	0.15 × 0.06 × 0.02 mm

### Data collection

**Table:**

XtaLAB Synergy, Dualflex, HyPix diffractometer	2006 independent reflections
Radiation source: micro-focus sealed X-ray tube, PhotonJet (Cu) X-ray Source	1927 reflections with *I* > 2σ(*I*)
Mirror monochromator	*R*_int_ = 0.050
Detector resolution: 10.0000 pixels mm^-1^	θ_max_ = 79.5°, θ_min_ = 4.5°
ω scans	*h* = −6→6
Absorption correction: multi-scan (CrysAlis PRO; Rigaku OD, 2021)	*k* = −11→10
*T*_min_ = 0.323, *T*_max_ = 1.000	*l* = −12→12
14165 measured reflections	

### Refinement

**Table:**

Refinement on *F*^2^	Hydrogen site location: inferred from neighbouring sites
Least-squares matrix: full	H-atom parameters constrained
*R*[*F*^2^ > 2σ(*F*^2^)] = 0.037	*w* = 1/[σ^2^(*F*_o_^2^) + (0.0617*P*)^2^ + 0.1479*P*] where *P* = (*F*_o_^2^ + 2*F*_c_^2^)/3
*wR*(*F*^2^) = 0.105	(Δ/σ)_max_ = 0.001
*S* = 1.09	Δρ_max_ = 0.37 e Å^−^^3^
2006 reflections	Δρ_min_ = −0.26 e Å^−^^3^
147 parameters	Extinction correction: SHELXL2018/3 (Sheldrick 2015b), Fc^*^=kFc[1+0.001xFc^2^λ^3^/sin(2θ)]^-1/4^
0 restraints	Extinction coefficient: 0.0061 (14)
Primary atom site location: dual	

### Special details

**Table:**

Geometry. All esds (except the esd in the dihedral angle between two l.s. planes) are estimated using the full covariance matrix. The cell esds are taken into account individually in the estimation of esds in distances, angles and torsion angles; correlations between esds in cell parameters are only used when they are defined by crystal symmetry. An approximate (isotropic) treatment of cell esds is used for estimating esds involving l.s. planes.

### Fractional atomic coordinates and isotropic or equivalent isotropic displacement parameters (Å^2^)

**Table:**

	*x*	*y*	*z*	*U*_iso_*/*U*_eq_	
F3	0.83669 (15)	0.51338 (8)	0.12281 (8)	0.0298 (2)	
F1	1.03655 (15)	0.72062 (9)	0.10632 (8)	0.0306 (2)	
F2	0.63947 (16)	0.69737 (10)	0.05569 (8)	0.0352 (2)	
O1	0.94235 (16)	0.85798 (10)	0.34833 (9)	0.0231 (2)	
N2	0.53525 (17)	0.75163 (11)	0.47862 (10)	0.0178 (2)	
N1	0.25848 (19)	0.55184 (12)	0.38082 (11)	0.0224 (2)	
C3	0.3285 (2)	0.66590 (13)	0.49404 (12)	0.0195 (3)	
C7	0.6410 (2)	0.87311 (13)	0.57981 (12)	0.0199 (3)	
H7	0.783693	0.930322	0.567582	0.024*	
C6	0.5364 (2)	0.90977 (14)	0.69840 (12)	0.0214 (3)	
H6	0.608051	0.993640	0.769395	0.026*	
C4	0.2206 (2)	0.70392 (14)	0.61568 (13)	0.0214 (3)	
H4	0.077951	0.646052	0.626999	0.026*	
C5	0.3224 (2)	0.82541 (14)	0.71850 (13)	0.0211 (3)	
C2	0.4210 (2)	0.56623 (14)	0.29402 (12)	0.0210 (3)	
H2	0.416403	0.500889	0.205342	0.025*	
C1	0.5986 (2)	0.68799 (13)	0.34799 (12)	0.0191 (3)	
C9	0.8297 (2)	0.66755 (14)	0.14284 (13)	0.0231 (3)	
C8	0.7991 (2)	0.74800 (13)	0.29223 (12)	0.0190 (3)	
C10	0.2118 (2)	0.87010 (16)	0.85047 (13)	0.0267 (3)	
H10A	0.336148	0.863035	0.927312	0.040*	
H10B	0.068569	0.799545	0.848113	0.040*	
H10C	0.158430	0.976875	0.862058	0.040*	

### Atomic displacement parameters (Å^2^)

**Table:**

	*U*^11^	*U*^22^	*U*^33^	*U*^12^	*U*^13^	*U*^23^
F3	0.0391 (5)	0.0189 (4)	0.0298 (4)	−0.0012 (3)	0.0086 (3)	−0.0001 (3)
F1	0.0318 (4)	0.0315 (4)	0.0291 (4)	−0.0052 (3)	0.0120 (3)	0.0047 (3)
F2	0.0349 (5)	0.0452 (5)	0.0241 (4)	0.0054 (4)	−0.0037 (3)	0.0081 (3)
O1	0.0223 (4)	0.0190 (4)	0.0270 (4)	−0.0048 (3)	0.0026 (3)	0.0037 (3)
N2	0.0161 (5)	0.0153 (5)	0.0222 (5)	−0.0015 (3)	0.0010 (4)	0.0058 (4)
N1	0.0197 (5)	0.0184 (5)	0.0280 (5)	−0.0036 (4)	0.0008 (4)	0.0044 (4)
C3	0.0158 (5)	0.0162 (5)	0.0272 (6)	−0.0019 (4)	0.0002 (4)	0.0079 (4)
C7	0.0180 (5)	0.0167 (5)	0.0241 (6)	−0.0029 (4)	−0.0006 (4)	0.0051 (4)
C6	0.0208 (6)	0.0192 (6)	0.0231 (6)	−0.0002 (4)	−0.0002 (4)	0.0044 (4)
C4	0.0177 (5)	0.0206 (6)	0.0282 (6)	−0.0006 (4)	0.0026 (4)	0.0110 (5)
C5	0.0201 (6)	0.0203 (6)	0.0250 (6)	0.0029 (4)	0.0023 (4)	0.0098 (5)
C2	0.0196 (6)	0.0180 (6)	0.0240 (6)	−0.0017 (4)	0.0000 (4)	0.0034 (4)
C1	0.0191 (6)	0.0167 (5)	0.0213 (6)	−0.0006 (4)	0.0008 (4)	0.0046 (4)
C9	0.0228 (6)	0.0225 (6)	0.0244 (6)	−0.0014 (4)	0.0025 (5)	0.0065 (5)
C8	0.0186 (5)	0.0162 (5)	0.0227 (6)	0.0010 (4)	0.0009 (4)	0.0063 (4)
C10	0.0278 (6)	0.0282 (7)	0.0264 (6)	0.0017 (5)	0.0054 (5)	0.0097 (5)

### Geometric parameters (Å, º)

**Table:**

F3—C9	1.3345 (14)	C6—H6	0.9500
F1—C9	1.3292 (14)	C6—C5	1.4245 (17)
F2—C9	1.3453 (14)	C4—H4	0.9500
O1—C8	1.2208 (15)	C4—C5	1.3728 (18)
N2—C3	1.3827 (14)	C5—C10	1.5028 (17)
N2—C7	1.3706 (15)	C2—H2	0.9500
N2—C1	1.4011 (15)	C2—C1	1.3987 (16)
N1—C3	1.3571 (16)	C1—C8	1.4247 (16)
N1—C2	1.3367 (16)	C9—C8	1.5489 (17)
C3—C4	1.3997 (17)	C10—H10A	0.9800
C7—H7	0.9500	C10—H10B	0.9800
C7—C6	1.3629 (17)	C10—H10C	0.9800
			
C3—N2—C1	106.60 (10)	N1—C2—C1	112.65 (11)
C7—N2—C3	122.01 (10)	C1—C2—H2	123.7
C7—N2—C1	131.39 (10)	N2—C1—C8	123.53 (11)
C2—N1—C3	105.23 (10)	C2—C1—N2	104.25 (10)
N2—C3—C4	119.63 (11)	C2—C1—C8	132.19 (11)
N1—C3—N2	111.28 (10)	F3—C9—F2	106.95 (10)
N1—C3—C4	129.10 (11)	F3—C9—C8	113.36 (9)
N2—C7—H7	120.8	F1—C9—F3	107.82 (10)
C6—C7—N2	118.35 (11)	F1—C9—F2	107.29 (10)
C6—C7—H7	120.8	F1—C9—C8	110.72 (10)
C7—C6—H6	119.2	F2—C9—C8	110.45 (10)
C7—C6—C5	121.61 (11)	O1—C8—C1	126.81 (11)
C5—C6—H6	119.2	O1—C8—C9	117.43 (10)
C3—C4—H4	120.2	C1—C8—C9	115.72 (10)
C5—C4—C3	119.55 (11)	C5—C10—H10A	109.5
C5—C4—H4	120.2	C5—C10—H10B	109.5
C6—C5—C10	119.98 (11)	C5—C10—H10C	109.5
C4—C5—C6	118.86 (11)	H10A—C10—H10B	109.5
C4—C5—C10	121.17 (11)	H10A—C10—H10C	109.5
N1—C2—H2	123.7	H10B—C10—H10C	109.5
			
F3—C9—C8—O1	130.29 (11)	C3—N1—C2—C1	0.16 (13)
F3—C9—C8—C1	−51.85 (14)	C3—C4—C5—C6	−0.07 (16)
F1—C9—C8—O1	8.98 (15)	C3—C4—C5—C10	179.86 (10)
F1—C9—C8—C1	−173.16 (10)	C7—N2—C3—N1	−179.78 (10)
F2—C9—C8—O1	−109.71 (12)	C7—N2—C3—C4	0.59 (16)
F2—C9—C8—C1	68.15 (13)	C7—N2—C1—C2	179.87 (11)
N2—C3—C4—C5	−0.35 (17)	C7—N2—C1—C8	−2.02 (19)
N2—C7—C6—C5	−0.07 (17)	C7—C6—C5—C4	0.29 (17)
N2—C1—C8—O1	1.14 (19)	C7—C6—C5—C10	−179.65 (10)
N2—C1—C8—C9	−176.48 (9)	C2—N1—C3—N2	−0.18 (13)
N1—C3—C4—C5	−179.91 (11)	C2—N1—C3—C4	179.41 (11)
N1—C2—C1—N2	−0.09 (13)	C2—C1—C8—O1	178.67 (12)
N1—C2—C1—C8	−177.96 (12)	C2—C1—C8—C9	1.04 (18)
C3—N2—C7—C6	−0.37 (16)	C1—N2—C3—N1	0.13 (13)
C3—N2—C1—C2	−0.03 (12)	C1—N2—C3—C4	−179.50 (9)
C3—N2—C1—C8	178.08 (10)	C1—N2—C7—C6	179.75 (11)

### Hydrogen-bond geometry (Å, º)

**Table:**

*D*—H···*A*	*D*—H	H···*A*	*D*···*A*	*D*—H···*A*
C2—H2···F3	0.95	2.53	2.9876 (14)	110
C4—H4···N1^i^	0.95	2.48	3.4139 (16)	167
C7—H7···O1	0.95	2.43	2.9864 (14)	117
C7—H7···O1^ii^	0.95	2.30	3.1464 (14)	147

Symmetry codes: (i) −*x*, −*y*+1, −*z*+1; (ii) −*x*+2, −*y*+2, −*z*+1.

## References

[bb1] Bagdi, A. K., Santra, S., Monir, K. & Hajra, A. (2015). *Chem. Commun.* **51**, 1555–1575. 10.1039/c4cc08495k 25407981

[bb3] Dhanalakshmi, G., Mala, R., Thennarasu, S. & Aravindhan, S. (2019). *IUCrData*, **4**, x191477.

[bb4] Farrugia, L. J. (2012). *J. Appl. Cryst.* **45**, 849–854.

[bb5] Fun, H.-K., Rosli, M. M., Kumar, D. J. M., Prasad, D. J. & Nagaraja, G. K. (2011). *Acta Cryst.* E**67**, o573. 10.1107/S1600536811003928 PMC3052008 21522335

[bb6] Gong, Y., Ma, H. & Li, J. (2012). *Acta Cryst.* E**68**, o1342. 10.1107/S1600536812013992 PMC3344477 22590239

[bb7] Gurbanov, A. V., Kuznetsov, M. L., Demukhamedova, S. D., Alieva, I. N., Godjaev, N. M., Zubkov, F. I., Mahmudov, K. T. & Pombeiro, A. J. L. (2020*a*). *CrystEngComm*, **22**, 628–633.

[bb8] Gurbanov, A. V., Kuznetsov, M. L., Mahmudov, K. T., Pombeiro, A. J. L. & Resnati, G. (2020*b*). *Chem. Eur. J.* **26**, 14833–14837. 10.1002/chem.202002518 32567710

[bb9] Guseinov, F. I., Malinnikov, V. M., Lialin, K. N., Kobrakov, K. I., Shuvalova, E. V., Nelyubina, Y. V., Ugrak, B. I., Kustov, L. M. & Mahmudov, K. T. (2022). *Dyes Pigments*, **197**, 109898.

[bb10] Khamees, H. A., Chaluvaiah, K., El-khatatneh, N. A., Swamynayaka, A., Chong, K. H., Dasappa, J. P. & Madegowda, M. (2019). *Acta Cryst.* E**75**, 1620–1626. 10.1107/S2056989019013410 PMC6829731 31709079

[bb11] Kopylovich, M. N., Mahmudov, K. T., Mizar, A. & Pombeiro, A. J. L. (2011). *Chem. Commun.* **47**, 7248–7250. 10.1039/c1cc11696g 21607242

[bb12] Koudad, M., Elaatiaoui, A., Benchat, N., Saadi, M. & El Ammari, L. (2015). *Acta Cryst.* E**71**, o979–o980. 10.1107/S2056989015021957 PMC4719933 26870561

[bb13] Ma, Z., Gurbanov, A. V., Maharramov, A. M., Guseinov, F. I., Kopylovich, M. N., Zubkov, F. I., Mahmudov, K. T. & Pombeiro, A. J. L. (2017*a*). *J. Mol. Catal. A Chem.* **426**, 526–533.

[bb14] Ma, Z., Gurbanov, A. V., Sutradhar, M., Kopylovich, M. N., Mahmudov, K. T., Maharramov, A. M., Guseinov, F. I., Zubkov, F. I. & Pombeiro, A. J. L. (2017*b*). *Mol. Catal.* **428**, 17–23.

[bb15] Ma, Z., Mahmudov, K. T., Aliyeva, V. A., Gurbanov, A. V., Guedes da Silva, M. F. C. & Pombeiro, A. J. L. (2021). *Coord. Chem. Rev.* **437**, 213859.

[bb16] Ma, Z., Mahmudov, K. T., Aliyeva, V. A., Gurbanov, A. V. & Pombeiro, A. J. L. (2020). *Coord. Chem. Rev.* **423**, 213482.

[bb17] Maharramov, A. M., Shikhaliyev, N. Q., Suleymanova, G. T., Gurbanov, A. V., Babayeva, G. V., Mammadova, G. Z., Zubkov, F. I., Nenajdenko, V. G., Mahmudov, K. T. & Pombeiro, A. J. L. (2018). *Dyes Pigments*, **159**, 135–141.

[bb18] Mahmudov, K. T., Gurbanov, A. V., Aliyeva, V. A., Resnati, G. & Pombeiro, A. J. L. (2020). *Coord. Chem. Rev.* **418**, 213381.

[bb19] Mahmudov, K. T., Huseynov, F. E., Aliyeva, V. A., Guedes da Silva, M. F. C. & Pombeiro, A. J. L. (2021). *Chem. Eur. J.* **27**, 14370–14389. 10.1002/chem.202102245 34363268

[bb20] Mizar, A., Guedes da Silva, M. F. C., Kopylovich, M. N., Mukherjee, S., Mahmudov, K. T. & Pombeiro, A. J. L. (2012). *Eur. J. Inorg. Chem.* **2012**, 2305–2313.

[bb21] Rigaku OD (2021). *CrysAlis PRO*. Rigaku Oxford Diffraction, Tokyo, Japan.

[bb22] Sheldrick, G. M. (2015*a*). *Acta Cryst.* A**71**, 3–8.

[bb23] Sheldrick, G. M. (2015*b*). *Acta Cryst.* C**71**, 3–8.

[bb24] Shikhaliyev, N. Q., Ahmadova, N. E., Gurbanov, A. V., Maharramov, A. M., Mammadova, G. Z., Nenajdenko, V. G., Zubkov, F. I., Mahmudov, K. T. & Pombeiro, A. J. L. (2018). *Dyes Pigments*, **150**, 377–381.

[bb25] Shikhaliyev, N. Q., Kuznetsov, M. L., Maharramov, A. M., Gurbanov, A. V., Ahmadova, N. E., Nenajdenko, V. G., Mahmudov, K. T. & Pombeiro, A. J. L. (2019). *CrystEngComm*, **21**, 5032–5038.

[bb26] Shixaliyev, N. Q., Gurbanov, A. V., Maharramov, A. M., Mahmudov, K. T., Kopylovich, M. N., Martins, L. M. D. R. S., Muzalevskiy, V. M., Nenajdenko, V. G. & Pombeiro, A. J. L. (2014). *New J. Chem.* **38**, 4807–4815.

[bb27] Spek, A. L. (2020). *Acta Cryst.* E**76**, 1–11. 10.1107/S2056989019016244 PMC6944088 31921444

[bb28] Tber, Z., Kansiz, S., El Hafi, M., Loubidi, M., Jouha, J., Dege, N., Essassi, E. M. & Mague, J. T. (2019). *Acta Cryst.* E**75**, 1564–1567. 10.1107/S2056989019012751 PMC6775750 31636995

[bb29] Turner, M. J., Mckinnon, J. J., Wolff, S. K., Grimwood, D. J., Spackman, P. R., Jayatilaka, D. & Spackman, M. A. (2017). *Crystal Explorer 17*. The University of Western Australia. http://hirshfeldsurface.net

